# Observations on Some Causes of the Excess of the Mortality of Males above That of Females

**Published:** 1788

**Authors:** Joseph Clarke

**Affiliations:** Physician to the Lying-in Hospital at Dublin


VI. *Obfervations on fome Caufes of the Excefs of the Mortality of Males above that of Females. By* Jofeph Clarke, *M. D. Phyfician to the Lying in Hofpital at Dublin. Communicated by the Rev.* Richard Price, *D. D. F. R. S. in a Letter to* Charles Blagden, *M. D. Sec. R. S.*—From the *Philofophical Tranfactions of the Royal Society of London.* Vol. LXXVI. *For the Year* 1786. Part. II. 4to. London. 1786.

Newington-Green, Feb. 6, 1786.

SIR,

I RECEIVED fome time ago the inclofed letters and regiftry from Dr. Clarke, Phyfician to the Lying-in Hofpital at Dublin. They contain fome accounts that feem to me not improper to be communicated to the Royal Society.

The obfervations which have been made on the laws that govern human mortality prove,

 that that the mortality of males exceeds that of females in almoſt all the ſtages of life, and particularly in the earlieſt ſtages; and that this exceſs prevails moſt in great towns, and all the leſs natural ſituations of human life. The facts in theſe papers throw ſome light on this ſubject. Male fœtus's requiring more nutrition than female fœtus's, becauſe larger, and being alſo for this reaſon more liable to injury in delivery, are brought into the world leſs perfect: and this happening more or leſs in proportion to the vigour and juſt formation of the mother, it muſt happen moſt in thoſe ſituations where the greateſt tenderneſs of frame and deviations from nature take place. The truth, in ſhort, ſeems to be, that any debility in either parent muſt affect moſt the production of that ſex which requires the largeſt and ſtrongeſt ſtamina; and that ſuch debilities prevailing moſt in great towns and poliſhed ſocieties, the exceſs of the mortality of males muſt alſo be greateſt in ſuch ſituations. And this I reckon the principal reaſon of a circumſtance in human mortality which before I received theſe communications from Dr. Clarke, I did not ſo well underſtand.

With much reſpect I am, &c.

RICH. PRICE.

*Dr.*

### Dr. Clarke's *first Letter to the Rev. Dr.* Price.

Dublin,

SIR,

IN your very useful Treatise on Life Annuities, &c. you remark *, that "it has been "observed, that the Author of nature has provided, that more males should be born "than females, on account of the particular "waste of males, occasioned by wars and other "causes. That perhaps it might have been "observed, with more reason, that this provision had in view that particular weakness "or delicacy in the constitution of males which "makes them more subject to mortality; and "which, consequently, renders it necessary "that more of them should be produced, in "order to preserve in the world a due proportion between the sexes." And further, you elsewhere remark †, that "the facts recited at "the end of your fourth Essay prove, that "there is a difference between the mortality of "males and females; but that you must however observe, that it may be doubted, whether "this difference so unfavourable to males, be

* Vol. I. p. 373. † Vol. II. p. 247.

"natural

"natural; and that there are facts which prove "that you have reason for such a doubt."* After stating a number of very satisfactory facts of this kind you remark, that "the inference "from them is very obvious; that they seem "to shew sufficiently, that human life in males "is more brittle than in females, only in consequence of adventitious causes, or of some "particular debility which takes place in polished and luxurious societies, and especially "in great towns."

What those adventitious causes are, or how this particular debility is produced and operates, are questions which appear to me highly interesting and curious. I have therefore been at considerable pains to examine and arrange a very accurate and extensive registry in such a manner as I hope will throw some light on these questions. As it is to the accuracy of modern registers that we are originally indebted for our knowledge of the facts in question, I apprehend, it is from the same source only that we shall be enabled satisfactorily to explain them.

Of the registry inclosed, I beg leave to observe to you, Sir, that it has been kept from its commencement by a man of uncommon accuracy (one of the under-clerks of the House of Commons); and that as the poor women and their children are obliged to paſs through his office, before leaving the Hoſpital, his ſituation is ſuch that there is no likelihood of his being deceived. It exhibits to our view the occurrences of twenty-eight years in above 20,000 inſtances: a number which I am inclined to think can hardly appear inſufficient for eſtabliſhing ſome general inferences and concluſions on a tolerably ſure foundation. Although my reaſoning on theſe matters ſhould not appear very concluſive, or my calculations perfectly accurate, yet I flatter myſelf, that the facts will neither be unacceptable nor uſeleſs to you.

I believe it may be ſafely aſſerted, that anatomy has not hitherto detected any internal difference between the animal œconomy of the male and female, which can be ſuppoſed to account for their difference of mortality, more eſpecially in early infancy; and this (it deſerves to be particularly remarked) is the period during which the chances are much the greateſt againſt male life. It is a matter of common obſervation that males, *cæteris paribus*, grow to a greater ſize than females, both *in utero* and every ſubſequent period of their growth. Conſequently,

ſequently, they muſt meet with more difficulty, and endure more hardſhip and fatigue, in the hour of birth. Accordingly, practitioners in midwifery, taught by experience, know, that when any conſiderable difficulty occurs in the birth of a child (for example, in all the different kinds of preternatural labours) they ſtand a much better chance of ſaving the life of a female than of a male. It is on this principle we can explain what our regiſtry concurs with others in proving, viz. that near one-half more males than females are ſtill-born. Naturaliſts are agreed, that the head of the human fœtus is larger in proportion to its body than that of any other animal; and I believe it is certain, that no animal whatever brings forth its young with ſo much difficulty, pain, and danger, as a woman. Now as we know that the head contains one of the moſt important organs of the body to life, it is highly reaſonable to ſuppoſe, that any additional injury which it ſuſtains in delivery may produce very material effects on the whole ſyſtem. Theſe effects though often may not be always immediate. They may operate in weakening the male conſtitution ſo as to render it more apt to be affected by any exciting cauſe of diſeaſe ſoon after after birth, and lefs able to ftruggle againft it. It may be afked, how this will apply to the difference of mortality in great towns and country fituations? The anfwer evidently is, that in great towns rickets, fcrophula, and other difeafes affecting the bones, and producing confequent mal-conformation of the female fex, are more frequent than in healthy country fituations.

There is another circumftance, Sir, which may have fome influence in producing that particular debility which you mention. It is this: as the ftamina of the male are naturally conftituted to grow to a greater fize, a greater fupply of nourifhment *in utero* will be neceffary to his growth than to that of a female. Defects in this particular, proceeding from delicacy of conftitution or difeafes of the mother, muft of courfe be more injurious to the male fex. And although the male children may be fo lucky as to efcape abortion and the perils of delivery, it is probable, that they will be more apt to languifh under difeafe, or die at fome future period, from the application of noxious caufes to an originally half-ftarved frame. To a perfon little accuftomed to confider phyfiological fubjects, this reafoning may appear fomewhat obfcure.

It may, perhaps, be somewhat illustrated by considering that nourishment of the fœtus *after* birth which nature has provided for. Suppose every mother in a great city obliged to suckle and nurse her own child, without the assistance of spoon-meat; and every mother in the adjacent country to do the same. Of the former there would not perhaps be one good nurse in five; and of the latter, perhaps, not one bad in ten. The difference of mortality that would ensue both to mothers and children thus situated, and the greater sufferings of the male than female sex, may be easily calculated. We see that, when a women conceives twins, and has two fœtuses *in utero* to nourish instead of one, it becomes peculiarly fatal both to her and her offspring. The chances are above four to one greater against her than against a woman bringing forth one child, and about two to one against her issue *.

Give me leave, Sir, to call your attention a little further to the facts relating to twins. They are singular and curious, at the same time that they serve to confirm some of the preceding

* Compare the 7th and 14th, 6th and 13th inferences in the annexed extracts.

reasoning.

reaſoning. Near *one-half* more twins die, and near *one-third* more are ſtill-born, than of ſingle children. And why?—It is not becauſe they meet with greater difficulties in the birth. On the contrary, it is a known fact, that, being much leſs than other children, women bring them forth with more eaſe. Does it not then proceed from a ſcanty nutrition, by which they are oftener blighted *in utero* than ſingle children; and, when born alive, have leſs ſtrength to ſupport life through the firſt ſtages of its exiſtence.

It is farther worthy of obſervation, that though *double* the numbers of twins die and are ſtill-born, compared to ſingle children, yet the proportion of male twins loſt to females is *leſs*. Only one-fifth more of the male ſex die than of the female, and only one-third more is ſtill-born. Whereas of ſingle children, whoſe proportional mortality is one-half leſs, *one-fourth* more of the male ſex die, and near double the number is ſtill-born. To what then are we to attribute this leſſened mortality in favour of male twins? Probably to their brain and nervous ſyſtem ſuffering leſs during delivery, on account of their heads being much ſmaller than thofe of fingle children. Were I difpofed to be prolix, I could offer many more plaufible arguments on this fubject; but to you, Sir, I am fure they would be unneceffary. There is only one circumftance remaining, relative to the proportion of the fexes, which I cannot pafs over in filence. We fee evident wifdom in the creation of a greater number of males than females; but why the proportion they bear to each other differs in different countries and fituations, and why there fhould be a feventeenth more males born of fingle children than twins, are queftions which I leave to be decided by thofe philofophers who underftand the theory of generation better than I do. Be this as it may, I am convinced that the majority in favour of the male fex is fooner deftroyed than the generality of writers feem to be aware of. Did the limits of this letter permit, I think, I could prove from Dr. Short's own data *, that the majority of males is deftroyed long before the common marriageable period; but I fhall content myfelf with an obfervation or two on the regiftry before us. If one-half of the whole born in this Hofpital die before three years, which is the eftablifhed com-

* New Obfervations, p. 72. & feq.

putation for great cities ; and if, on the loſs of ſomewhat more than a *third* of this half, a majority of 1177 be reduced to 483 by a loſs of 694, as appears from the regiſtry, it is pretty evident, that by the death of the two remaining thirds, a majority will be left in favour of the female ſex. It is obvious, that the ſtatement with regard to twins corroborates this ſuppoſition ; for of them, inſtead of a fifth, there is near one *half* dead and ſtill-born, the conſequence of which is, that we ſend out a majority of females. It may be objected, that their males do not bear ſo great a proportion to the females ; and that, therefore, it is not to be expected they ſhould keep up their majority ſo long. But there is only a ſeventeenth fewer males produced; whereas it has been already ſhewn, that there is a much greater proportion between the deaths of ſingle and twin males againſt the former and in favour of the latter.

Such are the outlines, Sir, of my ſentiments on this ſubject. I have aſſumed the liberty of addreſſing them to you without ceremony, as a well-wiſher to every member of the republic of letters. I ſhall be happy, ſhould your ſentiments happen to coincide with mine, or if I can be of any farther ſervice in promoting your very laudable inquiries.

I am, Sir, with great reſpect, &c.

JOSEPH CLARKE.

Lying-in Hoſpital,
June 9, 1785.

*Dr:* Clarke's *ſecond Letter to the Rev. Dr.* Price.

Dublin, Oct. 22. 1785.

SIR,

ENCOURAGED by your approbation of my former letter, I will take the liberty of ſtating to you a few more facts and obſervations, which I hope you will judge an appendix to it of ſome importance.

With the view of aſcertaining how far ſome of the foregoing conjectures are well founded, and of determining with greater preciſion the more obvious differences between the male and female ſex in infancy, I began in the month of July laſt by weighing forty children, twenty of each ſex, and by taking the dimenſions of their heads. In the months of Auguſt and September I repeated the ſame experiment twice, taking ſuch children as appeared to have arrived at the full full period of geſtation promiſcuouſly as they happened to be born.

I weighed them all a few hours after birth, before they had taken food, and before purgative medicines had time to operate. For this purpoſe, I made uſe of a ſmall ſpring or pocket ſteelyard, which weighs any thing (not heavier than a few pounds) appended to it with ſufficient accuracy. To this was attached a flannel bag, into which the children were put, at firſt, naked; but this I ſoon found troubleſome. The nurſes often wanted time ſufficient to aſſiſt me, and timid mothers were afraid of their infants catching cold; I was therefore obliged to weigh them with their cloaths on, and to ſubtract a certain quantity from the groſs weight of each child, according as it was full, middling, or light cloathed. Whatever inaccuracy this may have introduced, as to the real weight of the children, it can but little influence their comparative weights, or the differences between the two ſexes, which it was my object to aſcertain.

For meaſuring their heads, I made uſe of a piece of painted or varniſhed linen tape, divided into inches, halves, and quarters. The varniſh has the good effect of preventing the length of ſuch a meaſure being readily affected by variations tions in the humidity of the atmofphere, &c.; and it has little or no elafticity. In this part of the experiment then I can pretend to confiderable accuracy. I took firft the greateft circumference of the head from the moft prominent part of the occiput around over the frontal finufes; and, fecondly, the tranfverfe dimenfion from the fuperior and anterior part of one ear, acrofs the fontanelle, to a fimilar part of the oppofite ear. Thefe dimenfions appeared to me the moft likely to afford data for determining the refpective fizes of the brain in the different fexes. The refult was as follows:

| Twenty males. | | | Twenty females. | | |
|---|---|---|---|---|---|
| Weight. lbs. &c. | Circumference of heads. Inches. | Dimenfions from ear to ear Inches. | Weight. lbs. &c. | Circumference of heads. Inches. | Dimenfions from ear to ear. Inches. |
| Experiment 1. | | | | | |
| 149½ | 282 | 152 | 137¼ | 272 | 143 |
| Experiment 2. | | | | | |
| 144½ | 277 | 146¼ | 135 | 272 | 147 |
| Experiment 3. | | | | | |
| 248 | 280 | 147½ | 132 | 273 | 143¼ |
| Totals. | | | | | |
| 442 | 839 | 445¾ | 404¼ | 817 | 433¼ |
| Average weight, &c. | | | | | |
| 7lbs.5oz.7dr. | 14 | 7¼ | 6lbs.11oz.6dr. | 13⅝ | $7\frac{2}{9}$ |

Having

Having found the relative proportions between the fexes to turn out thrice with fo much uniformity, and obferving them to correfpond pretty nearly with fome experiments, made for very different purpofes by the late Profeffor Roederer, of Gottingen, I did not think it neceffary to profecute the fubject farther.

Upon the whole, it may be obferved, that the difference of weight between the male and female at birth may be rated at about nine ounces, or nearly a twelfth part of the original weight. In the circumference of their heads there is a difference of near half a inch, or about a 28th or 30th part; and the fame proportion of a 28th is pretty nearly preferved in the tranfverfe dimenfion. It is evident, as the bony paffage through which infants pafs is of a certain determined capacity, that, were their heads equally incompreffible with thofe of adults, the difference of half an inch in their fize would often prove fatal to them. By the compreffibility of their heads, however, in well formed women, this difficulty is by time furmounted. The effects which fuch a compreffion on the brain may produce, have not hitherto been well attended to.

 In

In reckoning children, weighing from $5\frac{1}{2}$ to $6\frac{1}{2}$, 6 pounds weight, and from $6\frac{1}{2}$ to $7\frac{1}{2}$, 7, and so forth, in order to avoid fractions, I find the number of males and females, arranged according to their weight, to stand as follow.

Males.

| lbs. | 4 | 5 | 6 | 7 | 8 | 9 | 10 |
|---|---|---|---|---|---|---|---|
| N° | 0 | 3 | 6 | 32 | 16 | 2 | 1 |

Females.

| lbs. | 4 | 5 | 6 | 7 | 8 | 9 | 10 |
|---|---|---|---|---|---|---|---|
| N° | 2 | 9 | 14 | 25 | 8 | 2 | 0 |

Hence it appears, that the majority of males runs thus: seven, eight, six, five; whilst that of the females is seven, six, five, eight. Hence also appears the merciful dispensations of Providence towards the female sex; for when deviations from the medium standard occur, it is remarkable that they are much more frequently below than above this standard. In 120 instances there are only five children exceeding eight pounds and a half in weight. The same may be observed with regard to the size of their heads. Only six measured above $14\frac{1}{2}$ inches in circumference, and these all of the male sex; five measured $14\frac{3}{4}$, and one 15. In transverse dimensions only four exceeded $7\frac{3}{4}$, the largest of which was $8\frac{1}{2}$; whereas deviations under the standard n these particulars were very numerous numerous, never however under 12 around and 6¼ acrofs.

In the year 1753, Dr. Roederer publifhed a Paper, *De pondere & longitudine Infantum recens natorum*, in the Commentaries of the Royal Society of Gottingen, of which the celebrated Haller was the principal inftitutor, and long the prefident. In this Paper he proves, in the cleareft manner, by inconteftible experiments, the abfurdity of the ideas of obftetric writers with regard to the progrefs of the ovum during geftation, and the weight of the fœtus after birth. He fhews, although they ftate the weight of the fœtus, come to the full time, to be from 12 to 14 or 16 pounds, that it is more generally 6 or 7, and very rarely exceeds 8. This deferves particular notice for two reafons; firft, becaufe it ferves to fhew how little dependence is to be placed on the affertions of authors who copy each other fervilely, without having recourfe to experiment even in the moft obvious cafes; and, fecondly, becaufe this paper has been overlooked by fome of the moft celebrated writers and teachers of midwifery now living. What idea are we to form of the accuracy of one of our lateft fyftematic writers, who (telling us that he has been a practitioner of midwifery, in a capital city, for twenty years, and a teacher for more than twelve) ftates, in one page of his work, that the weight of a fœtus at eight months is about feven pounds; and on the oppofite page, that at full time it weighs from twelve to fourteen pounds*?

Of twenty-feven children, carried to the full period of geftation, weighed and meafured in length by Roederer, without any attention to the difference of fex, I find, that eighteen were of the male and nine of the female fex; and that the average weight of the former was about 6 lbs. 9 oz., that of the latter about 6 lbs. 2 oz. 2 dr. Whether he and I ufed the fame weights, I cannot exactly fay. He obferves, that he ufed the civil pound of Gottingen, which I can eafily perceive confifted of 16 ounces, as mine did; but whether a German ounce be the fame with ours, I have not *data* to determine. The average length of the males

* See a Treatife of Midwifery (pages 88. and 89.) divefted of technical terms and abftrufe theories, by A. Hamilton, M. D. 8vo. edit. London, 1781.

meameafured by him is about $20\frac{1}{3}$ inches, and of the females about $19\frac{7}{18}$. He weighed alfo the placentæ of 21 lying-in women, 16 of whom had borne male children, and five female. The average weight of the former was 1 lb. $2\frac{1}{2}$ oz.; that of the latter 1 lb. 2 oz. Hence it appears, that in other circumftances, befides thofe I have taken notice of, the male and female fex differ. So far I thought it neceffary to take extracts from Dr. Roederer's paper, as his obfervations and mine throw light on each other, and add confirmation to both.

The limits of this letter will not permit me, Sir, to trefpafs much farther on your patience. There is one circumftance or two fo intimately connected with my former letter, that I cannot pafs them over in filence. Having found that males fuffer more in the birth than females, I was defirous of knowing whether the chance of the mother's recovery was thereby in any degree affected; and to determine this I was once more at the pains of turning over our regiftry with care. I found, that of 214 women, dead of fingle children, 50 were delivered of ftill-born males, and 15 of ftill-born females; 76 of living males, and 73 of living females.

females. Of the 15 dead of twins, 6 had twins one of each sex; 6 others had twins both of the male sex; and three had twins both of the female sex. All of which twins (two or three excepted), it is very remarkable, survived the death of their mothers. It would appear then, that the life of the mother is principally endangered in those cases where the bulk of the male's head precludes the possibility of his being brought into the world alive, either by the efforts of nature or art. The conception of twins we have observed to be more fatal to the mother than that of single children. The average weight of 12 twins, which have occurred to me of late, I find to be 11 lbs. a pair. The largest pair weighed 13 lbs. and the least 8½. From some rude attempts made to ascertain the weight of the contents of the gravid uterus in cases of twin and single children, I am inclined to think, that they are to each other as about 15 to 10, or perhaps 14½ to 9¼.

Believe me, Sir, with great respect, &c.

J. CLARKE.

### An Abstract of the Registry kept at the Lying-in Hospital, in Dublin,

### From the 8th of December, 1757, to the 31ſt of December, 1784.

### By B. H. Regiſter.

| | | Number of Patients admitted. | Went out not delivered. | Delivered in the Hoſpital. | Boys born. | Girls born. | Total number of children. | Women having twins. | Children dead. | Children ſtill-born. | Women dead. |
|---|---|---|---|---|---|---|---|---|---|---|---|
| From 8th to 31ſt December, - - - | 1757 | 55 | — | 55 | 30 | 25 | 55 | — | 6 | 3 | 1 |
| | 1758 | 455 | 1 | 454 | 255 | 207 | 462 | 8 | 54 | 21 | 8 |
| | 1759 | 413 | 7 | 406 | 228 | 192 | 420 | 13 | 95 | 22 | 5 |
| | | | | | | | | 1 had 3 | | | |
| | 1760 | 571 | 15 | 556 | 300 | 260 | 560 | 4 | 116 | 36 | 4 |
| | 1761 | 537 | 16 | 521 | 283 | 249 | 532 | 11 | 104 | 29 | 9 |
| | 1762 | 550 | 17 | 533 | 279 | 266 | 545 | 12 | 106 | 33 | 6 |
| | 1763 | 519 | 31 | 488 | 274 | 224 | 498 | 12 | 94 | 29 | 9 |
| | 1764 | 610 | 22 | 588 | 287 | 308 | 595 | 7 | 83 | 28 | 12 |
| | 1765 | 559 | 26 | 533 | 288 | 251 | 539 | 6 | 94 | 25 | 6 |
| | 1766 | 611 | 30 | 581 | 324 | 261 | 585 | 4 | 111 | 18 | 3 |
| | 1767 | 695 | 31 | 664 | 373 | 301 | 674 | 10 | 125 | 29 | 11 |
| | 1768 | 689 | 34 | 655 | 362 | 302 | 664 | 9 | 154 | 47 | 16 |
| | 1769 | 675 | 33 | 642 | 350 | 301 | 651 | 9 | 152 | 38 | 8 |
| | 1770 | 705 | 35 | 670 | 372 | 305 | 677 | 7 | 107 | 37 | 8 |
| Year ending 31ſt of December, - - | 1771 | 724 | 29 | 695 | 370 | 341 | 711 | 16 | 102 | 44 | 5 |
| | 1772 | 725 | 21 | 704 | 368 | 344 | 712 | 8 | 116 | 32 | 4 |
| | 1773 | 727 | 33 | 694 | 367 | 344 | 711 | 17 | 136 | 31 | 13 |
| | 1774 | 709 | 28 | 681 | 357 | 334 | 691 | 10 | 154 | 29 | 21 |
| | 1775 | 752 | 24 | 728 | 364 | 378 | 742 | 14 | 122 | 27 | 5 |
| | 1776 | 883 | 31 | 802 | 418 | 407 | 825 | 22 | 132 | 39 | 7 |
| | | | | | | | | 1 had 3 | | | |
| | 1777 | 872 | 37 | 835 | 452 | 395 | 847 | 12 | 145 | 35 | 7 |
| | 1778 | 961 | 34 | 927 | 476 | 460 | 936 | 9 | 127 | 39 | 10 |
| | 1779 | 1064 | 53 | 1011 | 550 | 476 | 1026 | 15 | 146 | 59 | 8 |
| | 1780 | 967 | 48 | 919 | 499 | 441 | 940 | 21 | 115 | 41 | 5 |
| | 1781 | 1079 | 42 | 1027 | 598 | 447 | 1045 | 18 | 121 | 38 | 6 |
| | 1782 | 1021 | 31 | 990 | 549 | 458 | 1007 | 17 | 127 | 57 | 6 |
| | 1783 | 1230 | 63 | 1167 | 632 | 553 | 1185 | 17 | 91 | 72 | 15 |
| | | | | | | | | 1 had 3 | | | |
| | 1784 | 1317 | 57 | 1260 | 642 | 640 | 1282 | 23 | 76 | 68 | 11 |
| Totals | | 20625 | 839 | 19786 | 10647 | 9470 | 20117 | 331 | 3111 | 1006 | 229 |

Proportion of males and females born, about *nine* males to *eight* females.

children dying under *ſixteen* days old, as *one* to about *ſix and a half*.

children ſtill-born, as *one* to about *twenty*.

women having twins, as *one* to about *ſixty*.

women dying in child-bed, as *one* to about *eighty-ſeven*.

### Extracts from the Registry kept at the Lying-in Hospital, Dublin, from the Year 1757 to 1784.

### Uniparous.

| Women. Delivered in Hospital. | Women. Dead. | Children. Sex. M. | Children. Sex. F. | Children. Dead. M. | Children. Dead. F. | Children. Still-born. M. | Children. Still-born. F. |
|---|---|---|---|---|---|---|---|
| 19455 | 214 | 10305 | 9150 | 1656 | 1247 | 602 | 351 |
| | | 9150 | | 1247 | | 351 | |
| | | 19455 | | 2903 | | 953 | |
| | | | | 953 | | | |
| | | | | Total 3856 dead and still-born. | | | |

### Multiparous, Twins, Triplets, &c.

| Women. Delivered in Hospital. | Women. Dead. | Children. Sex. M. | Children. Sex. F. | Children. Dead. M. | Children. Dead. F. | Children. Still born. M. | Children. Still born. F. |
|---|---|---|---|---|---|---|---|
| 331 | 15 | 342 | 320 | 116 | 91 | 29 | 20 |
| | | 320 | | 91 | | 20 | |
| | | 662 | | 207 | | 49 | |
| | | | | 49 | | | |
| | | | | Total 256 dead and still-born. | | | |

Inferences.

1. Proportion of males to females born nearly as - 17 to 15

2. ——— children dying under 16 days - 1 to $6\frac{2}{3}$

3. ——— children still-born - - 1 to $20\frac{2}{5}$

4. ——— males dying to females - - 4 to 3

5. ——— —— still-born to ditto - - 12 to 7

6. ——— still-born and dead of each sex to the whole 1 to 5

7. ——— women dying in child-bed - - 1 to 92

Inferences.

8. Proportion of male twins to females born - - 17 to 16

9. ——— twins dying under 16 days - - 1 to $3\frac{1}{5}$

10. ——— twins still-born - - - 1 to $13\frac{1}{2}$

11. ——— male twins dying to females - - 5 to 4

12. ——— —— still-born to ditto - 3 to 2

13. ——— still-born and dead of each sex to the whole 1 to $2\frac{3}{5}$

14. ——— women dying - - - 1 to 22

Totals of dead and still-born.

| | Males. | Females. |
|---|---|---|
| | 1656 | 1247 |
| | 602 | 351 |
| | 2258 | 1598 |
| Born in hospital | 10305 | 9150 |
| Dead and still-born | 2258 | 1598 |
| Sent out living | 8047 | 7552 |
| | 7552 | |
| Balance - - | 495 in favour of the male sex. | |

Totals of dead and still-born, whether uniparous or multiparous.

| | Males. | Females. |
|---|---|---|
| | 1656 | 1247 |
| | 116 | 91 |
| | 602 | 351 |
| | 29 | 20 |
| | 2403 | 1709 |
| Born | 10647 | 9470 |
| | 2403 | 1709 |
| | 8244 | 7761 |
| | 7761 | |

Totals of twins, &c., dead and still-born.

| | Males. | Females. |
|---|---|---|
| | 116 | 91 |
| | 29 | 20 |
| | 145 | 111 |
| Born - - | 342 | 320 |
| Dead and still-born | 145 | 111 |
| Sent out living - | 197 | 209 |
| | | 197 |
| Balance in favour of the female sex | | 12 |

Of 20117 children born, at the end of a fortnight, there is only a balance of 483 in favour of the male sex, although originally 1177; greater loss of males 694.

